# MRI in the Evaluation of Locally Advanced Vulvar Cancer Treated with Chemoradiotherapy and Vulvar Cancer Recurrence: The 2021 Revision of FIGO Classification and the Need for Multidisciplinary Management

**DOI:** 10.3390/cancers14163852

**Published:** 2022-08-09

**Authors:** Maura Miccò, Luca Russo, Salvatore Persiani, Miriam Dolciami, Lucia Manganaro, Teresa Margarida Cunha, Catarina Janicas, Stefania Rizzo, Olivera Nicolic, Giorgia Garganese, Luca Tagliaferri, Valentina Lancellotta, Giovanni Scambia, Riccardo Manfredi, Benedetta Gui

**Affiliations:** 1 Dipartimento Diagnostica per Immagini, Radioterapia Oncologica ed Ematologia, Fondazione Policlinico Universitario A. Gemelli, IRCCS, 00168 Rome, Italy; 2 Department of Radiological, Oncological and Pathological Sciences, Sapienza University of Rome, 00185 Rome, Italy; 3 Department of Radiology, Instituto Portugues de Oncologia de Lisboa Francisco Gentil, 1099-023 Lisbon, Portugal; 4 Department of Radiology, Centro Hospitalar de Lisboa Ocidental, 1349-019 Lisbon, Portugal; 5 Faculty of Biomedical Sciences, University of Italian Switzerland (USI), 6900 Lugano, Switzerland; 6 Service of Radiology, Imaging Institute of Southern Switzerland, Clinica Di Radiologia EOC, 6900 Lugano, Switzerland; 7 Center of Radiology, Clinical Center of Vojvodina, Faculty of Medicine, University of Novi Sad, 21000 Novi Sad, Serbia; 8 Gynecology and Breast Care Center, Mater Olbia Hospital, 07026 Olbia, Italy; 9 Dipartimento di Scienze della Vita e Sanità Pubblica, Università Cattolica del Sacro Cuore, 00168 Rome, Italy; 10 UOC di Radioterapia Oncologica, Dipartimento Diagnostica per Immagini, Radioterapia Oncologica e Ematologia, Fondazione Policlinico Universitario A. Gemelli IRCCS, 00168 Rome, Italy; 11 UOC Ginecologia Oncologica, Dipartimento per la Salute della Donna, del Bambino e di Sanità Pubblica, Fondazione Policlinico Universitario A. Gemelli, IRCCS, 00168 Rome, Italy; 12 Dipartimento Universitario di Scienze Radiologiche ed Ematologiche, Università Cattolica del Sacro Cuore, 00168 Rome, Italy

**Keywords:** MRI, female pelvic MRI, vulvar anatomy, vulvar cancer

## Abstract

**Simple Summary:**

Vulvar cancer is a rare gynecologic tumor (representing 4% of all gynecologic malignancies). We review the role of MRI in patients with locally advanced vulvar cancer (LAVC), highlighting the findings that influence clinical management. We also present the MRI findings of local recurrence according to its type and location.

**Abstract:**

Magnetic resonance imaging (MRI) plays an essential role in the management of patients with locally advanced vulvar cancer (LAVC), who frequently benefit from a multidisciplinary approach. Accordingly, chemoradiotherapy (CRT) with radical or neoadjuvant intent seems to provide a better quality of life and less morbidity than extensive surgery alone. In this overview, we discuss the role of MRI in the post-CRT assessment of LAVC, emphasizing the evaluation of primary tumor response. In order to assess treatment response and select candidates for post-CRT local excision, the MRI findings are described according to signal intensity, restricted diffusion, enhancement, and invasion of adjacent organs. We also focus on the role of MRI in detecting vulvar cancer recurrence. It occurs in 30–50% of patients within two years after initial treatment, the majority appearing near the original resection margins or in ipsilateral inguinal or pelvic lymph nodes. Finally, we describe early and delayed complications of CRT, such as cellulitis, urethritis, vulvar edema, bone changes, myositis, and fistulization. By describing the role of MRI in assessing LAVC response to CRT and detecting recurrence, we hope to provide suitable indications for a personalized approach.

## 1. Introduction

Vulvar cancer is a rare gynecologic tumor, representing 4% of all gynecologic malignancies [1]. Approximately one-third of patients with vulvar carcinoma are diagnosed with locally advanced disease at presentation [2]. This has severe implications for patient survival, as 5-year survival rates range from 86% for localized disease to 53% for locally advanced disease, and 19% for patients with distant metastases [3]. However, there is little consensus regarding the definition of the best initial therapeutic approach.

Historically, the term LAVC has been used to describe different clinical scenarios, including: (a) large primary tumors (>4 cm) invading adjacent perineal organs; (b) tumors presenting with bulky inguinal lymphadenopathies, which may be fixed to the fascia, muscle, or vessels; and (c) tumors that cannot be managed with radical vulvar resection [4]. A staging system has been proposed by the International Federation of Gynecology and Obstetrics (FIGO), and was revised in 2021 (Table 1). According to this system, stages IIIA and IVA are considered LAVC [5].

The baseline evaluation of vulvar cancer is based on imaging investigations that are chosen according to the first clinical examination. This is the first approach to assess the extent of disease, providing information about the number of vulvar lesions and the relative site and size, the distance to midline, and finally, the presence of palpable inguinal lymph nodes. A concurrent ultrasound of the inguinal lymph nodes provides more accurate information about lymph node status [6,7].

When at clinical examination, the surrounding perineal tissues or medial structures are infiltrated. Additional imaging is essential to assess the involvement of the deep organs such as the vagina, urethra, bladder, anal canal, and rectum, and specifically, MRI is recommended given the high soft tissue resolution [8,9].

Additional investigations could be required as appropriate, such as endoscopy (cystoscopy/ano–rectoscopy), to evaluate the mucous membranes of infiltrated pelvic organs, and CT or FDG PET/CT to assess the involvement of pelvic lymph nodes and distant organs [10,11,12].

Therefore, a combined clinical and imaging evaluation is essential in addressing the specific individualized treatment approach. Currently, this may include radical surgery with or without plastic reconstruction, external beam radiotherapy (RT), chemoradiotherapy (CRT), and interventional radiotherapy (IRT, Brachytherapy), which may be used in combination [13,14,15] or with other local therapies, such as electrochemotherapy [16]. The chosen approach depends not only on tumor extent, but also on the patient’s performance status and clinical characteristics. In fact, patients with LAVC are often older women with age-associated comorbidities [17,18,19]. Because more than 50% of patients develop severe post-surgical complications, sometimes motivating additional procedures, the frequently required radical surgery is not normally recommended in these patients [20]. Adjuvant RT or CRT may follow, depending on residual disease and the risk of recurrence [15,21]. However, there is no robust evidence on the benefit of neoadjuvant CRT and RT, which can be explained by small sample sizes, study heterogeneity, and divergent CRT and RT protocols [22]. Several groups have also evaluated definitive CRT by comparing the results obtained from patients treated with neoadjuvant CRT. Compared to upfront radical surgery, definitive CRT (+/−IRT) allows organ preservation with good clinical outcomes and no significant differences in patient survival [23]. Curative treatment may not be possible due to the advanced stage of disease or poor patient performance status. Moreover, local RT can be used to achieve symptom palliation and improve quality of life [24]. IRT may also be useful, particularly in patients with severe comorbidities and contraindications to surgery [18,25]. The technique allows the delivery of a high radiation dose to the tumor, while sparing the adjacent perineal organs. Research is currently focused on intensity-modulated RT for VC [1,26], which maximizes the radiation dose to the target volume, reducing toxicity and exposure to adjacent organs. Target therapies are also under development [13].

MRI plays an essential role in contemporary RT planning due to its high soft tissue resolution and tumor delineation, as well as its ability to detect the invasion of adjacent structures [9,27,28]. Moreover, assessment of tumor response is a key factor in defining the appropriate approach after CRT. Although MRI is generally accepted in the evaluation of tumor response after multimodal treatment, there is no sufficient supporting evidence in the literature. When there is no evidence of residual disease, clinical observation is recommended. On the contrary, patients with residual disease must be considered for tumor resection or additional RT/CRT [1,14,25,26]. Therefore, the multidisciplinary tumor board is crucial in defining the best approach for each patient with LAVC, either before or after multimodal treatment [8,14,25,29].

In this pictorial review, we will discuss the role of MRI for patients with LAVC who are candidates for CRT, and describe the MRI features of LAVC during treatment and follow-up, highlighting the findings that influence clinical management. In addition, we will address the MRI features of local recurrence according to its type and location.

## 2. MRI Technique

The currently accepted MRI protocol for patients with VC has been proposed by the European Society of Urogenital Radiology (ESUR) (Table 2) [9]. Recommendations for optimal patient preparation include fasting 4–6 h before the examination, anti-peristaltic agent administration, and bladder voiding; vaginal opacification with gel is optional. The patient should be imaged supine, with a phased array pelvic coil [11] or an eight-channel cardiac coil [12].

## 3. MRI Findings of LAVC Prior to CRT

MRI is considered the best imaging technique with which to perform local staging [9,27]. VC manifests as a mass of intermediate-to-high signal intensity on T2-weighted images (WI), with avid enhancement and restricted diffusion. According to the new updated 2021 FIGO staging system, stage I tumor is confined to the vulva; stage II tumor extends to the lower one-third of the vagina and/or of the urethra, and the anus; stage III tumor involves the adjacent perineal structures (upper two-thirds of the vagina and/or urethra, bladder or rectal mucosa; IIIA) or not fixed, nonulcerated inguinal lymph nodes (IIIB and IIIC). Differently, stage IV is defined by the extension to the pelvic bone (IV A) or the presence of distant metastasis, including pelvic lymph nodes (IV B) [5].

In order to ease communication between radiologists and referring physicians, and to allow for accurate treatment planning, we propose a point-by-point MRI report.

### 3.1. Vagina

LAVC extension to the vagina manifests as a disruption to the vaginal wall caused by the intermediate-to-high signal intensity mass on T2-WI [10], with simultaneous loss of the linear appearance of the vaginal vault. Obliteration of the paravaginal fat is also seen when the tumor extends beyond the vagina. Though coronal and sagittal T2-WI are essential in depicting the extension of vaginal invasion, contrast-enhanced imaging and diffusion-weighted imaging (DWI) increase diagnostic accuracy; vaginal opacification with gel may improve the visualization of small tumors [12].

### 3.2. Urethra

The female urethra has a characteristic target-like appearance on axial T2-WI, consisting of four concentric rings (mucosa, submucosa, muscularis, and serosa). In the setting of urethral invasion, this target appearance is disrupted by the intermediate-to-high signal intensity mass [8,11].

### 3.3. Anal Sphincter Complex

The most significant components of the anal sphincter are the levator ani and puborectal muscles. The former is a funnel-shaped muscle extending from the obturator muscle to the anal canal, while the latter is located at the insertion of the levator ani muscle onto the anal canal. Anal sphincter invasion is considered when the shortest distance between the tumor and the muscle is less than 3 mm, or when gross invasion is seen.

### 3.4. Bladder and Rectum

**Figure 1:**
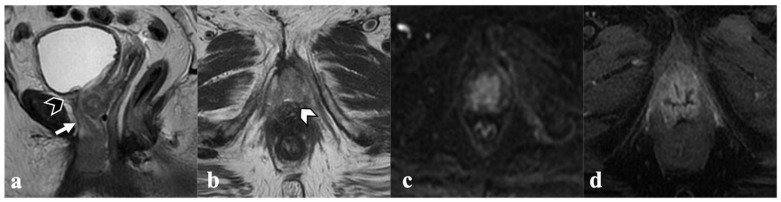
Locally advanced vulvar cancer. A 78-year-old patient with bulky tumor infiltrating both labia minora and majora (not shown), anteriorly, the urethra ((**a**)—white arrows) and the urinary bladder neck ((**a**)—black arrowhead), and cranially, the middle third of the vagina ((**b**)—white arrowhead). Mass also shows diffusion restriction (**c**) and contrast enhancement (**d**).

MRI has a high diagnostic accuracy in excluding bladder or rectal invasion in patients with gynecological cancers, reaching negative predictive values of 96–100% [30]. Bladder and rectal invasion are best depicted on sagittal T2-WI, defined by disruption of the high signal intensity mucosal layer and obliteration of the fat planes (Figure 1).

Likewise, DWI and contrast-enhanced imaging increase diagnostic accuracy, showing the presence of an area of restricted diffusion and/or abnormal enhancement in the bladder or rectal wall, or the direct extension of soft tissue into the bladder or rectum.

### 3.5. Inguinal or Pelvic Nodes

A general recommendation is to use a short-axis threshold of 10 mm to identify metastatic lymph nodes on MRI. The loss of fatty hilum and ovoid shape, rounded contour, and irregular borders should raise suspicion for nodal involvement from tumor. Furthermore, lymphadenopathies may exhibit heterogeneous signal intensity on T2-weighted and on contrast-enhanced images due to central necrosis. DW imaging can aid in nodal detection, since lymph nodes may be easily identified by their high signal intensity. By combining functional and structural information, FDG PET/CT has improved sensitivity for identifying metastatic lymph nodes [8,10,11,12].

## 4. Post-CRT MRI Findings

As previously discussed, neoadjuvant CRT reduces the need for radical surgery in patients with LAVC. Only 5% of patients with FIGO 2009 stages III and IV tumors show residual unresectable disease after treatment, with almost 45% of patients having no macroscopic residual disease [31,32]. Moreover, Shylasree et al. have shown no significant differences in survival compared to primary surgery [33]. Therefore, post-CRT MRI assessment is essential in determining the need for additional therapy.

Clinical assessment and a follow-up MRI are usually performed four and six weeks after finishing CRT, respectively. The MRI report must include the elements necessary to restage VC: local tumor status, residual invasion of adjacent organs, and lymph node status. To avoid potential pitfalls, the acquisition must range from the mons pubis to the inguinal region. The vulva must be centered, and the oblique planes adequately oriented. A comparative evaluation between pre- and post-CRT studies is also recommended in order to accurately interpret the imaging findings and restage the tumor. In particular, radiologists should carefully address the original tumor location and its extension into the adjacent organs, and compare T2-WI, DWI, and post-contrast images site by site.

### 4.1. Local Tumor Status and Residual Invasion of Adjacent Organs

**Figure 2:**
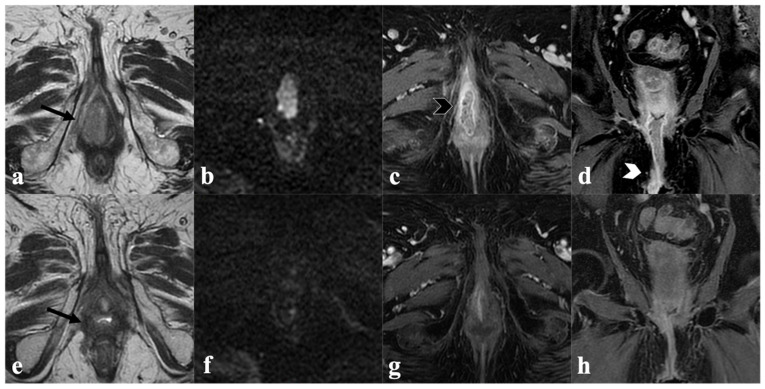
Complete response to CRT. An 85-year-old patient with locally advanced vulvar cancer (FIGO stage IIIA) treated with CRT, showing complete response to treatment. Baseline MRI (upper row) with axial T2-weighted ((**a**)—black arrow) axial DWI (**b**) post-contrast fat-saturated axial (**c**) and coronal (**d**) T1-weighted images shows infiltrative vulvar tumor involving, anteriorly, the clitoris and urethral meatus (black arrowhead in (**c**)), posteriorly, the fourchette, and cranially, the lower third of vaginal introitus (white arrowhead in (**d**)). MRI after CRT (bottom row) shows absence of a T2 intermediate signal intensity mass ((**e**)—black arrow), no hyperintensity in DWI (**f**), and no enhancement in post-contrast images (**g**,**h**) consistent with complete response.

Radiologic complete response is defined by the absence of a T2 intermediate-signal-intensity mass, which may be replaced by a T2 low signal intensity area, in keeping with post-treatment fibrosis. In this setting, no areas of restricted diffusion are seen in the prior location of the tumor (Figure 2).

**Figure 3:**
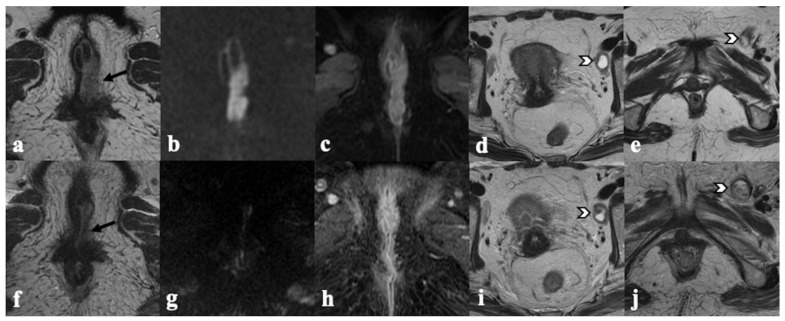
Partial response after CRT. A 73-year-old patient with locally advanced vulvar cancer (FIGO stage IVA) presenting with an intermediate T2 signal intensity mass involving left labia minor and major ((**a**)—black arrow), with hyperintensity in DWI (**b**) and enhancement in T1-WI post-contrast (**c**) and ulcerated pelvic ((**d**)—white arrowhead) and left inguinal ((**e**)—white arrowhead) lymph nodes. MRI after CRT (bottom row) shows significant lesion size reduction in both T2W ((**f**)—black arrow), DWI (**g**) and post-contrast T1W images (**h**), but dimensional increase in regional lymph nodes ((**i**,**j**)—white arrowheads), consistent with partial response.

Meanwhile, partial response is depicted as a combination of tumor reduction and a decrease in signal intensity (Figure 3).

Differentiating a residual tumor from post-treatment fibrosis is not always straightforward. In fact, radiologists tend to overestimate residual disease when evaluating T2-WI in isolation, partly due to post-treatment edema. Nevertheless, the residual tumor tends to exhibit a nodular appearance, while fibrosis is presented with spiculated linear margins. Contrast-enhanced imaging once again increases diagnostic accuracy, as only the residual tumor will enhance avidly, with nodular appearance. DWI has also been shown to be helpful in the post-CRT assessment of gynecological cancers, depicting tumor viability and necrosis before these changes are visible on morphologic acquisitions [34]. Oblique axial T2-WI and DWI sequences must be correctly orientated to better evaluate the anatomy of the vulva, vagina, and urethra, and to determine the presence of a residual tumor versus post-CRT findings. In fact, post-CRT findings such as focal thickness of the skin or superficial vulvar part, and focal or diffuse thickness of the vagina or urethral wall, may mimic residual disease on morphological images. Careful combined evaluation of DWI and post-contrast images is also very useful in discriminating pathology versus post-CRT findings. Moreover, fused T2-WI and DWI images are another key to evaluating post-CRT MRI to better discover small residual foci in the treated vulvar region.

In addition to the current tumor measurements, residual invasion of adjacent organs must be thoroughly described to provide the oncologist with the information required to plan further treatment. The urethra and urethral meatus may be carefully assessed, since persistence of their involvement may preclude surgery or require anterior pelvic exenteration. In the same manner, persistence of anus/rectal infiltration may preclude surgery or necessitate a posterior pelvic exenteration. Moreover, persistence of pelvic bone and/or adjacent pelvic muscles usually exclude surgery.

### 4.2. Lymph Node Status

Similar to the primary tumor, CRT may reduce malignant lymph nodes in both size and number, with up to 40% of patients submitted to CRT presenting with nodal downstaging [31]. Parallel to the dimensional criteria reported above, morphologic criteria assessed on T2WI may be used for nodal assessment after CRT. After CRT, suspicious nodes may still show heterogeneous internal signal intensity, often similar to that of the viable primary tumor and irregular margins. However, it is important to keep in mind that MRI cannot detect microscopic residual disease.

Chest and abdominal CT, as well as PET-CT, also play an important role in assessing the lymph nodes status and distant metastases.

### 4.3. Structured MRI Report

In order to ease the communication, we recommend using a structured MRI report comparing pre- and post-CRT images, which includes:

- Residual tumor location (right, left, central, bilateral);
- Residual tumor size (three-dimensional measurements);
- Comparative evaluation of tumor T2 signal intensity, restricted diffusion, and degree of enhancement;
- Residual invasion to adjacent organs (urethra, vagina, anus, rectum, pelvic sidewall);
- Presence of post-treatment fibrosis;
- Lymph node assessment;
- Description of the remaining pelvic organs.

## 5. Vulvar Cancer Recurrence

Recurrence consists of tumor reappearance after a disease-free interval of at least six months, most frequently occurring within the first two years of follow-up after the initial treatment. International guidelines (ESGO, NCCN) recommend symptom assessment and physical examination of the vulva and inguinal region every 3–6 months during the first two years, and every 6–12 months in the fourth and fifth years [1,35]. Afterward, predisposition to gynecological cancer must be taken into account. Any suspected recurrent or persistent disease should be biopsied. Imaging evaluation is indicated when there is a clinical suspicion of recurrence.

**Figure 4:**
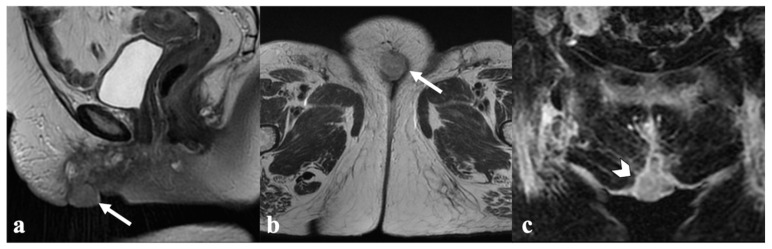
Recurrence on mons pubis. A 68-year-old patient previously treated with anterior vulvectomy for squamous cell carcinoma of the clitoris, presents 7 months later with intermediate T2 signal intensity mass in the subcutaneous tissue of mons pubic ((**a**,**b**)—white arrow) showing enhancement in post-contrast T1 coronal image ((**c**)—white arrowhead), consistent with recurrence.

VC recurrence may occur in the vulva, inguinofemoral lymph nodes, or distant sites. In more than 50% of patients, the recurrence takes place near the surgical resection margins (Figure 4).

**Figure 5:**
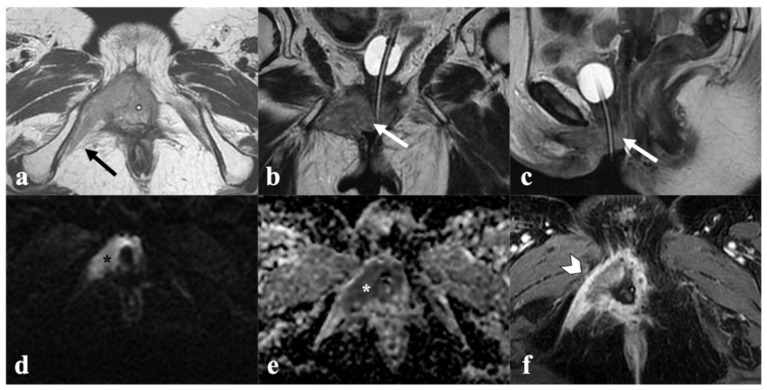
Skin bridge recurrence. A 68-year-old patient previously treated with radical vulvectomy, lymphadenectomy, and RT for locally advanced vulvar carcinoma, presenting 5 years later with a voluminous mass of intermediate T2 signal intensity infiltrating, laterally, the right internal obturator muscle ((**a**)—black arrow), cranially, the lower third of the vagina ((**b**)—white arrow), and anteriorly, the urethra ((**c**)—white arrow). The solid tissue shows diffusion restriction in both DWI ((**d**)—black asterisk) and ADC map ((**e**)—white asterisk) and marked enhancement in post-contrast T1-WI ((**f**)—white arrowhead), consistent with recurrence.

Inguinal node recurrence occurs in 22% of cases, most frequently in patients with initial lymph node involvement [36]. In patients previously submitted to surgery, recurrence may rarely occur in the skin bridges located between the prior vulva and the ipsilateral inguinal region (Figure 5).

Once more, initial lymph node involvement constitutes a risk factor [37].

Accordingly, we perform an ultrasound evaluation of the inguinal region, including the mound and the skin bridges. Any lesions that raise suspicion of recurrent or residual disease are biopsied in order to obtain histological confirmation.

**Figure 6:**
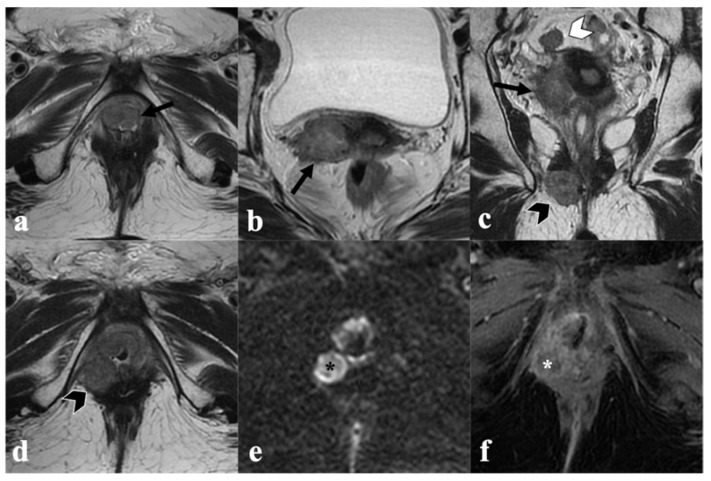
Locally advanced recurrence with satellite nodules. A 66-year-old patient previously treated with excisional biopsy and RT for squamous cell carcinoma of the right labium minus, presenting 3 years later with multiple solid pelvic nodules of intermediate T2 signal intensity, the largest in the lower III of vagina ((**a**,**d**)—black arrow), attached to the right lateral uterine wall ((**b**,**c**)—black arrow), in pelvic peritoneum ((**c**)—white arrowhead) and in the ischio-anal fossa ((**c**,**d**)—black arrowhead). Satellite nodules also show diffusion restriction ((**e**)—black star key) and peripheral enhancement ((**f**)—white star key).

In MRI, local recurrence is depicted by the presence of a new lesion, most frequently infiltrating adjacent perineal structures. Its signal intensity is similar to that of the initial lesion and is also associated with restricted diffusion and early avid enhancement, which increase diagnostic sensitivity in small lesions [9] (Figure 6).

**Figure 7:**
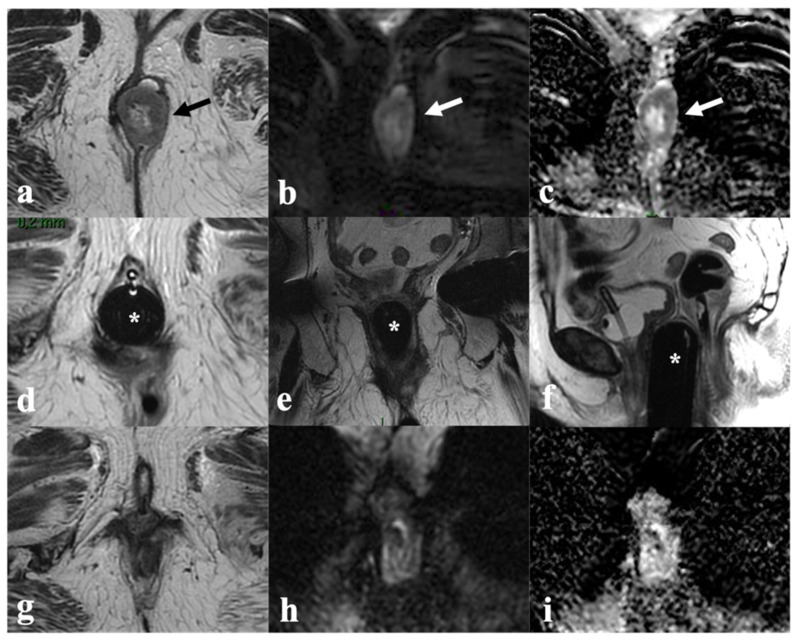
Complete response to CRT and brachytherapy in recurrent vulvar cancer. An 80-year-old patient with recurrent vulvar cancer after radical vulvectomy and lymphadenectomy, presenting with intermediate T2 solid tissue in left vulvar region ((**a**)—black arrow) with restricted diffusion in DWI and ADC map ((**b**,**c**)—white arrow). After CRT, brachytherapy was performed, with MRI monitoring after applicator insertion ((**d**–**f**)—white star key); axial (**d**) coronal (**e**) and sagittal (**f**) T2 images show that the applicator is correctly positioned, and no residual disease is visible. MRI at treatment completion shows tumor disappearance in both T2WI (**g**) and DWI/ADC map (**h**,**i**).

**Figure 8:**
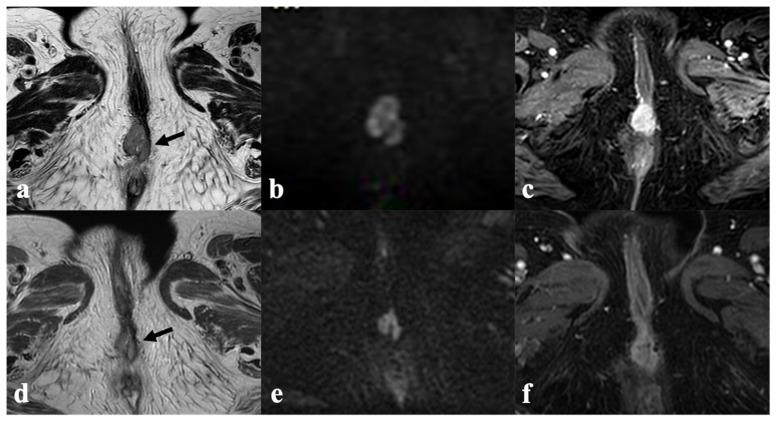
Partial response to RT, in recurrent vulvar cancer. A 79-year-old patient with recurrent vulvar cancer after partial vulvectomy (upper row) presenting with an intermediate T2 signal intensity mass in the right vulvar region ((**a**)—black arrow), with hyperintensity in DWI (**b**) and enhancement in T1-WI post-contrast (**c**). MRI after CRT (bottom row) shows lesion size reduction in both T2W ((**d**)—black arrow), DWI (**e**) and post-contrast T1W images (**f**) consistent with partial response.

As previously discussed, comparison with previous studies, DWI, and contrast-enhancement imaging help to allow for the correct diagnosis. Cutaneous bridge recurrences can occur in patients treated surgically, with the recurrent disease centered in the surgical inguinal and/or vulvar margins, extending to the adjacent structures. A description of the site and the dimensions of the local recurrence and the adjacent involved structures are essential for further planning decisions. Moreover, the MRI findings must be integrated with those obtained from chest and abdominal CT and from PET-CT (assessment of distant metastases) in order to determine the therapeutic approach. Wide radical local excision and inguinal lymphadenectomy have shown cure rates of 70%. Meanwhile, in a select group of patients with central recurrence invading the perineal organs, pelvic exenteration (anterior, posterior, or total) may be indicated with curative intent. A combination of CRT (if never performed) and radical local excision has also shown excellent results, with improved survival and reduced morbidity in advanced vulvar recurrence [38] (Figure 7 and Figure 8).

## 6. Post-Therapy Complications

Chemotherapy can lead to neutropenia and sepsis.

The potential complications from RT may be classified as early or late. Early complications include gastro/urogenital and skin disorders, lower limb lymphedema, and vulvar edema. On the other hand, late effects comprise myositis, insufficiency fractures, fistulization, and, in rare cases, radiation-induced tumors.

RT-induced myositis is caused by focal muscle degeneration. Recognition of its benign nature is paramount, as it can mimic residual or recurrent disease. In the same way as muscle edema, myositis shows increased T2 signal intensity and correlates to the RT field [29].

**Figure 9:**
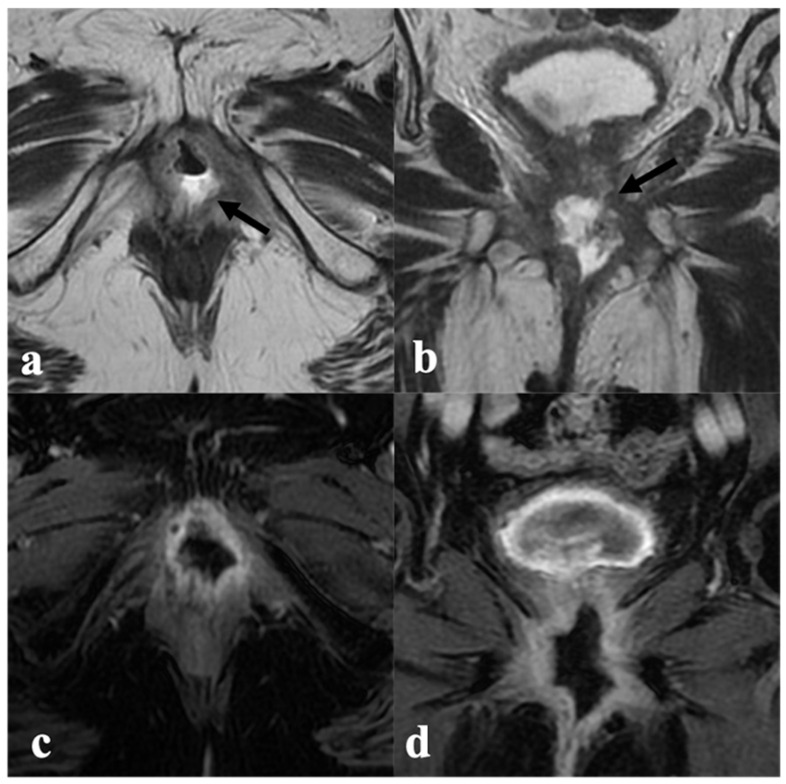
Urethrovaginal fistula after CRT. An 87-year-old patient who received multiple treatments (both surgery and CRT) for recurrent vulvar cancer, presenting with tissue loss in the urethrovaginal space resulting in a fluid-filled communication between the urethra and the lower third of vagina ((**a**,**b**)—black arrow) with marked peripheral enhancement (**c**,**d**), consistent with a fistula.

**Figure 10:**
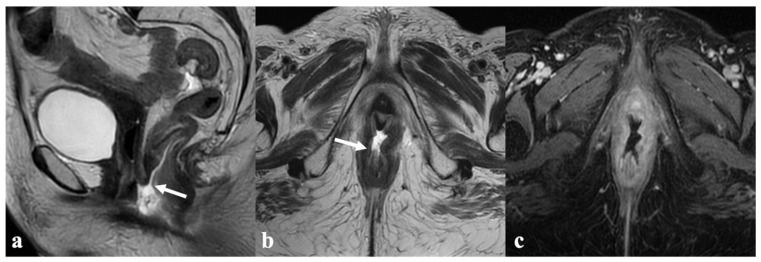
Anovaginal fistula after CRT. A 58-year-old patient treated with CRT for locally advanced vulvar cancer (FIGO stage IVA), presenting with a fluid-filled anovaginal fistulous tract connecting the lower vagina and the anal canal ((**a**,**b**)—white arrow), with marked peripheral enhancement in post-contrast T1-WI (**c**).

RT may also lead to diffuse bladder and rectal wall thickening, which is associated with increased T2 signal intensity. However, it shows neither avid enhancement nor restricted diffusion, in contrast to tumor invasion. RT-related injury may also cause fistulization, namely rectovaginal, entero-vaginal, and vesicovaginal fistulas, which typically manifest during the first year. MRI is accurate in the detection and characterization of fistulas, showing a T2 hyperintense tract with scattered foci of low signal intensity, in keeping with air bubbles. In the presence of active inflammation, the tract will also show peripheral enhancement (Figure 9 and Figure 10).

The use of multiplanar MR images allows for the complete delineation of fistulas. The sagittal plane is preferred for assessing rectovaginal and vesicovaginal fistulas, as it clearly depicts both the posterior bladder wall and anterior rectal wall disruption [21].

## 7. Conclusions

The invasiveness of surgery in patients with LAVC remains a concern. CRT (+/−IRT) has recently emerged as a viable alternative, considering the morbidity associated with extensive surgical procedures. Pre-CRT MRI plays a key role in treatment planning in this setting, since it depicts tumor extension, degree of adjacent organ invasion, and suspicious lymph nodes. On the other hand, post-CRT MRI is essential in assessing tumor response and determining the need for further treatment and follow-up. After a disease-free interval of at least six months, vulvar carcinoma recurs in many patients within the first two years. Most recurrences occur locally or in inguinal and pelvic lymph nodes. In addition to detecting vulvar cancer recurrence, MRI is also helpful in assessing potential CRT-related complications.

Therefore, radiologists should be familiar with the MRI findings, staging, and management of LAVC, in order to accurately assess tumor response and detect recurrence.

## Figures and Tables

**Table 1:** FIGO staging for carcinoma of the vulva: 2021 revision.

Stage	Definition
I	Tumor confined to the vulva.
IA	Tumor size ≤ 2 cm and stromal invasion ≤ 1 mm
IB	Tumor size > 2 cm or stromal invasion > 1 mm
II	Tumor of any size with extension to lower one-third of the urethra, lower one-third of the vagina, lower one-third of the anus with negative nodes
III	Tumor of any size with extension to upper part of adjacent perineal structures, or with any number of nonfixed, nonulcerated lymph node
IIIA	Tumor of any size with disease extension to upper two-thirds of the urethra, upper two-thirds of the vagina, bladder mucosa, rectal mucosa, or regional lymph node metastases ≤ 5 mm
IIIB	Regional lymph node metastases > 5 mm
IIIC	Regional lymph node metastases with extracapsular spread
IV	Tumor of any size fixed to bone, or fixed, ulcerated lymph node metastases, or distant metastases
IVA	Disease fixed to pelvic bone, or fixed or ulcerated regional lymph node metastases
IVB	Distant metastasis

**Table 2:** MRI protocol.

CE-DW-MRI
Imaging Protocol
- T1-weighted fast spin-echo (FSE) sequence in axial plane - T2-weighted FSE sequences in two imaging planes (axial/sagittal) - Small FOV T2-weighted FSE sequences in axial oblique plane (perpendicular to the long axis of the urethra) - Small FOV T2-weighted FSE sequences in oblique coronal plane (parallel to the long axis of the urethra) - DWI using a single-shot spin-echo echo-planar imaging sequence with high b-values (0, 1000 s/mm^2^) with the same orientation and location used to acquire axial oblique FSE T2-weighted images - T2-weighted FSE sequence in axial plane up to renal hila - Three-dimensional spoiled gradient-pulse FS T1 weighted before and after administration of 0.1 mmol/kg of gadolinium at a rate of 2 mL/sec, followed by a 20 mL saline bolus injection, in the axial/axial oblique plane and in the coronal/coronal oblique plane, pre- and post-contrast administration, after an 18 s of delay and at 15 s intervals for 3 scans to better obtain arterial portal and equilibrium phases
